# Some unusual forms of early onset postpartum psychosis

**DOI:** 10.1007/s00737-016-0676-7

**Published:** 2016-10-16

**Authors:** Ian Brockington

**Affiliations:** University of Birmingham, Edgbaston, Birmingham,, B15 2TT UK

**Keywords:** Postpartum delirium, Postpartum stupor, Unusual cyclical disorders, Bipolar disorder, Cycloid episodes

## Abstract

In addition to bipolar cycloid episodes, infective delirium and eclamptic psychosis, each of which has characteristic clinical features and course, brief episodes of delirium and stupor are also seen in the immediate aftermath of parturition. Several mothers have had similar episodes developing later in the first 10 days, and some have cyclical disorders with an unusual time base. Bipolar/cycloid disorders can start on day 1 or even earlier.

## Introduction

The first week or two after childbirth is the phase when the common forms of postpartum psychosis begin, including bipolar/cycloid episodes, infective delirium and eclamptic psychoses. These have well-defined features such as the manic or acute polymorphic syndrome, sepsis and seizures; but they also have a typical duration—about 2 months for non-organic episodes, and 2 weeks for eclamptic psychoses, with septic psychoses intermediate (Brockington 2014; Brockington 2016). This paper will describe several forms of postpartum psychosis with a different psychopathology and duration. It will also discuss the very early onset of bipolar/cycloid episodes.

## Postpartum delirium

During labour, there are more than 50 cases of unexplained delirium, first described in the eighteenth century (Kirkland 1774). These were well recognized in the early nineteenth century, but became rare after the introduction of effective analgesia. They were thought to be associated with painful delivery, but this case of postpartum delirium was described (Barth 1828):

A 24-year old German woman, pregnant for the first time, gave birth to a healthy boy after a 13-hour labour. Ten minutes later, the placenta was delivered, immediately followed by strong after-pains returning every 4–8 minutes. She began to rave and rage. She recognized none of those present (not even her husband), hit out violently, tried to get out of bed and could only be held down by several attendants. She said she was being threatened by murderers and thieves and shouted for help. She kicked out at the child when it was brought to calm her. She cursed her husband, with whom she lived in harmony. The uterus felt as hard as a stone. The doctor prescribed opium and castor oil. She gradually became quieter, and fell asleep. After a few hours, she woke completely restored, and had no memory for these events.

This was also associated with pain, but there are about 20 other cases in the literature, without mention of pain. Two had recurrent disorders, including this mother, who had a single episode after one birth and a phasic disturbance after another (Vanden Bosch 1880):

Immediately after her 5th child was born, a 30-year old mother suffered a *délire* with cries and agitation; this settled down after two hours. With her 6th pregnancy she had a severe and prolonged labour and became agitated. After delivery she had a postpartum haemorrhage; she was given some ergot and the uterus contracted. Suddenly she was seized by *délire*, with agitation and incessant movements. The memory of a deceased relative came to her mind, and she spoke as if this person was present. She was exasperated by the efforts made by the doctor, the nurse and her husband to restrain her, and became violent. She cried out, wept and demanded why she was not allowed to visit this dead person. This lasted 4–5 minutes, after which she seemed to wake, and take note of where she was. But, after a pause, it all started again with the same manifestations, but was this time more violent and persistent. She had nine more attacks in two hours. The penultimate attack occurred four hours after the birth, and then she fell asleep. Eleven hours after delivery she had another attack lasting 30 minutes. She slept for seven hours and remained well. She awoke with no memory for these events.

Amnesia was mentioned in nine cases. Severe blood loss (Weiskorn 1897) may have been a factor in one, and anaesthetics or other drugs (Manton 1892; Bourson 1958; Weinger et al 1988; Davis et al 1996) in four cases. As for frequency, Neubürger (Kirchberg 1913) had seen only case of this kind in 3160 births, and Engelhard (Engelhard 1912) had one possible case in nearly 20,000 deliveries. Thus, it is less common than parturient confusion.

## Postpartum stupor

In the early nineteenth century, two papers appeared describing the onset of stupor immediately after the infant was born. The first (Kelso 1840) described two cases of ‘nervous exhaustion’ seen in his practice of 1000 deliveries, one of which (described below) was recurrent:

A 38-year old English mother gave birth to her 7th child, after labour lasting 20 hours; she had received an oral dose of laudanum. After delivery of the placenta she complained of headache and a crushing sensation in the lower chest. Ten minutes later she was speechless and apparently dying. She was in profound stupor. Her eyes remained partly open and fixed, or the lids drooped languidly showing some portion of the eyeballs. Respiration was imperceptible, the heart regular but weak. The countenance was wan and expressionless, the skin pallid and cold. The limbs were feeble and lifeless, but retained for a short time the position in which they were placed. From this state of alarming prostration, the patient could be aroused, with difficulty and only temporarily, by pinching, bellowing in the ears, volatile salts, burning feathers applied to the nostrils or cold water. After half an hour she briefly rallied, then relapsed. A series of episodes continued, diminishing in severity and duration, with each recovery more complete. When she was fully recovered, four hours later, she was ‘affrighted and wondered what in all the world could have occasioned such an assemblage of anxious relatives and friends’. She had only a feeble recollection of what occurred since her delivery. Another obstetrician who attended two previous confinements said she had similar attacks on each occasion.

Under the name of *melancholia attonita*, a German author (Tott 1844) described three similar stupors, of which this is one:

A 20-year old German mother, whose sister suffered from puerperal melancholia, gave birth to her first child. Immediately after delivery she lay motionless as if struck by lightning. Her eyes were open and she appeared to be lost in amazed contemplation, as if unable to grasp the immensity of the extraordinary event that had happened to her. Entreaty, shouting and shaking had no effect. With open eyes she seemed to see nothing, hear nothing and feel nothing. She took something to drink without a murmur. There was no catalepsy. She recovered in 24 hours.

These transitory states of impaired consciousness, with no apparent organic cause, are similar to those seen during labour, which can occasionally persist after the birth, as in this example (Snoeck 1902):

A 28-year old Belgian mother felt her first labour pains in the evening. The midwife advised rest and she fell asleep. At 1 am, her husband was awakened by a sigh. He called his wife, then shook her, but there was no response. The midwife was unable to wake her and called the doctor. Physical examination was normal. Her pupils reacted to light. A cold wet towel and smelling salts had no effect. A hot iron and needle prick produced only a slight movement of the arm. Labour progressed without any sign of pain. The infant was born at 7 am, with no change in her condition. She remained ‘asleep’ for three days, without eating, or passing water or stool, then awoke. It was difficult to convince her she had been a mother for so long. She had no memories since the onset of labour, and had even forgotten a visit to a neighbour that afternoon. Her second birth was normal.

These stupors are marked by their close association with parturition itself, but other transitory disturbances have been reported later in the first ten postpartum days. The next case is unique, recurrent and familial, with organic features; it may have been an as-yet undescribed metabolic disorder (Sieche and Giedke 1999):

A 26-year old German mother suddenly became agitated and aggressive four days after her first delivery. She had uncontrolled movements, did not recognize familiar people, was disorientated and could only repeat single words in a stereotyped way. This lasted for 24 hours, with amnesia for the episode. Four years later she gave birth to her 2nd child. In the evening of day 2 she developed involuntary movements of her mouth and arms. She talked in a bewildered way, was disorientated in time, place and situation, did not recognize her husband and confused the names of her daughters. She recovered the next morning, with partial amnesia for the episode. The electro-encephalogram showed generalized slowing with delta waves. Her twin sister developed the same complication after her 2nd child was born: on day 3 she became confused, disorientated, agitated and aggressive. On day 4 she had seizures and lapsed into coma. The electro-encephalogram showed generalized slowing. A computerised tomographic scan showed cerebral oedema. On day 5 she died and necropsy showed necrosis of the pituitary and ischaemic lesions especially in the hippocampus.

Another transitory stupor started on day 7, and lasted less than 24 h (Steinberger 1831):

A 22-year old German mother gave birth to her 1st child. On day 6 she imprudently stood on the cold floor (in February) with bare feet, and started shivering. On day 7 she became immobile, staring in front of her, looking confused. The doctor arrived to find her standing stiff and upright, staring at her hands. She responded to questions by a melancholic gaze. She began to improve after 12 hours, and recovered the next day.

Two similar cases have been reported (Dörschlag 1886; Dretler 1930):

A 17-year old German mother gave birth to her 1st child. On day 4 she suddenly became confused and tried to throw the child out of the window, because it had a white head. From that moment she sat quite still, stared in front of her all day and took no notice of her surroundings. She was admitted to hospital, and it was not possible to get a word out of her. Three days later she recovered and could not understand how she came to try to jettison her child. She remained well thereafter.

A 36-year-old Polish mother developed impaired consciousness 2 days after a difficult delivery and responded to questions only with a vague gesture. She had stereotypic movements. In hospital, she became excited at times, shouted and recited the Lord’s Prayer at the top of her voice, saw devils, and heard the voice of her dead mother. After 2 days, she recovered but 24 h later had another brief period of excitement.

Lest it be thought that these disorders are only of historic interest, a London mother consulted me because she felt aggrieved about her obstetric and psychiatric management. She described two similar episodes occurring on days 4 and 9:

A strong and determined, thoughtful and caring, well-educated 35-year old, holding down a responsible job, became pregnant for the 1st and only time. At 42 weeks gestation, after a 10-hour labour, she was delivered by emergency forceps (for foetal distress), of a baby weighing 8 lb 8 oz. In spite of epidural anaesthesia, the pain was ‘insane’ and continuous. She required blood transfusions, and suffered from perineal discomfort due to an infected episiotomy wound, but without fever. On day 3 (75 hours after the birth), she felt ‘quite creative’. “I had all these ideas for paintings. I started drawing, and writing ideas on the back of a paper towel”. Later that morning she entered a dream-like state. “There was a wall of ice between me and the world. I felt paralysed, and could only move my arms”. She could hear people talking but was unable to answer. She could not understand why she was unable to move or speak, and had a sense of doom. “The main thing I felt was complete fear. I thought I had died, and maybe this is what death is like - an in-between world”. Her husband described her as ‘stiff and statuesque, on a frieze, eyes open but not talking, somewhere else’. She was incontinent of urine, and disorientated in time and space. As a mark of the severity of her disorder, the obstetric team ordered a brain scan. On the next day she awoke perfectly well, and remembered the experience as a bad dream. She was transferred to the psychiatric hospital. By this time she had a painful perineal suture, haemoglobin 7.6 g/100 ml and leucocyte cell count 16.3/mm^3^ with 87 % neutrophils. Six days later she had a second episode: she lapsed into a trance. “I thought they would section my husband and were poisoning me. I was in a floating state, unable to get up off the bed”. All night she could not move, lying rigid. In the morning, she returned to normal. A community midwife diagnosed the perineal infection (now accompanied by fever), and alerted the obstetric team. She was transferred to the women’s hospital and recovered after antibiotic treatment.

In this case, there was a differential diagnosis of infective delirium, but her infection was milder than the puerperal sepsis associated with organic psychosis, and the clinical picture was different.

## Unusual cyclical disorders

Puerperal bipolar/cycloid episodes often follow a relapsing course, with a time base of about a month (Brockington in press), but this mother had a different time base (Meschede 1903):

A German mother became ill in the first postpartum week with a 5-day cycle of acute confusion and *Tobsucht* [raving], followed by a day of sleep and normal behaviour. Fourteen months later she was admitted to the Königsberg asylum. The alternating course continued for 26 months, after which she recovered. Ten years later she had a recurrence and was readmitted for another 18 months, again with a cyclical course, first on a 3-day, then 4-day and then 5-day basis, by which time she had one day of *Tobsucht*, one day of sleep and three days rest.

Another mother had 10 relapses on a short time base during the early puerperium (Bennewitz 1837):

A 30-year old was delivered of her 1st child by forceps. On day 3 she became euphoric and garrulous. On day 12 she was anxious and restless, sensing that her end was near. This passed off, but returned the next evening (1st relapse). She recovered and was well for some days. On day 18 she had a recurrence of anxiety, euphoria and confused ideas: laughter and crying rapidly succeeding each other (2nd relapse). On day 20 she had a 3rd relapse, this time with delusions that followed each other like lightning; it lasted 2–3 hours. On day 22 she had a 4th relapse, taking the form of religious themes and nocturnal restlessness, followed by exhaustion. On day 24 she had her 5th relapse, with *Tobsucht.* The 6th relapse on day 26 was the worst: she let out a stream of obscenities, which (a modest woman) she had never spoken before, and scratched and bit anyone within reach. The 7th relapse on day 28 was milder, taking the form of a tiresome humour, with less confused thinking. The 8th relapse on day 30 was mild - she had a sense of pressure below, as if she was about to give birth. The 9th relapse on day 32 and the 10th and last on day 34 were no more than an abnormal chattiness and cheerfulness.

Two mothers have developed a diurnal pattern (Hattingen 1838; Dickson 1870), of which this is one:

A 30-year old gave birth. On day 3 she suddenly fell into frightful raving: she no longer wanted to know her husband and child, leapt out of bed, smashed a window, tore her clothes and tried to destroy everything in the room. The next day she lapsed into stupor, out of which she could only be wakened with difficulty. In the first 14 days this switching from disturbance to calm appeared at irregular intervals, and then developed an intermittent pattern, in which attacks of raving started every evening at 11 pm and lasted until daybreak. With treatment, stupor changed to sleep, but the raving continued. She was treated with opium and quinine, and, after an unstated time, recovered.

## Very early onset of bipolar/cycloid episodes

A study of the onset of 792 of early postpartum episodes reported in the literature, and 155 from my own series, showed no clear mode in the first ten days, a fall on day 11 and steep fall on day 15 with hardly any episodes after the 14th day (Brockington. 2014). There were 29 cases in the literature and 17 in my series with onset on day 1, a frequency about half that on days 2–10.

Onset during parturition was first described in 1847 (Macdonald 1847):

A 28-year old American mother complained of headache and depression during the last month of her 4th pregnancy. Towards the end she became sleepless and excited about religious subjects and in great anxiety about her confinement [prodromal symptoms, but insufficient evidence of prepartum psychosis]. In the 1st stage of labour, her mind was wandering; she talked incoherently on religious subjects, imitating the Quaker tone of preaching. As labour failed to progress, she became more and more delirious. A putrefied infant was born. She slept for a time, and awoke in a wild state of mind, and attempted to jump out of the window. She became so violent that it was necessary to fasten her to the bed. On day 6 she was raving uninterruptedly and incoherently, repeatedly using indecent words. By day 7 she had less than six hours sleep since the birth. She improved, but 17 days after the birth relapsed and was removed to an asylum.

There are 13 other cases in the literature, and four in my own series, as in this example:

A woman was reared by a paranoid mother in a noisy and chaotic home. At the age of 24 she had the first of two cycloid episodes. At 27 she gave birth to her only child @ 42 weeks gestation. During her 36-hour labour, she misidentified a student midwife as an acquaintance from a religious cult. She could feel insanity coming on. “I got very paranoid, thinking that staff were saying negative things about me”. Within hours she was ‘dipping in and out of psychosis’. After the birth she became confused and frightened. By day 3 she was staring blankly, not speaking, refusing food, drink and medication, and incontinent of urine. By day 8 she improved, as if awaking from a bad dream. A week later she relapsed, but with six ECT again recovered within two weeks. In the course of 24 years she had five unrelated episodes, with a variety of diagnoses including hypomania, paranoid psychosis and schizophreniform disorder.

Allowing 1 day for parturition, the frequency is about half that on day 1, but higher than prepartum, post-abortion or late postpartum onset groups. Three had a relapsing pattern, three had previous psychotic episodes and two had other postpartum episodes.

There are also instances of onset immediately *before* labour, as in this case (Sivadon 1933):

A 19-year old, with a history of ‘dementia praecox’, developed confusion, terrifying onirism, anxiety and mutism in the 3rd trimester. The next day she gave birth. Nine days later she was admitted to hospital.

Three mothers in my series had similar experiences: one suffered two cycloid episodes, which started 2 days after, and 3 days before her children were born (Brockington et al. 1990). Another, with several other episodes after the birth or miscarriages, had an episode starting the day before she went into labour.

One could conclude that, beyond doubt, early postpartum bipolar/cycloid episodes can start on day 1, and, on the balance of probability, during labour. Onset before labour is speculative. Onsets are important when searching for causes—in this case the triggers of bipolar/cycloid episodes. Onset during labour, or even on day 1, precedes the endocrinological changes initiated by lactation. Parturient onset, when the placenta is still *in situ*, is perhaps too early for the postpartum cascade of sex steroids and chorionic gonadotropic hormone. It suggests a trigger related to the onset of labour.

## Discussion

These uncommon cases are important to clinical practice and research. They appear to be rare, but might be less rare if their existence was recognized. Mothers have a right to advice from consultants with a comprehensive knowledge of the psychoses of childbearing, not just the most common. In research it is important to focus on homogeneous groups, and the inclusion of postpartum delirium and stupor introduces unnecessary heterogeneity. The limits of onset of postpartum bipolar/cycloid disorders are important in the identification of the trigger.
